# 3D Reconstruction of the Neurovascular Unit Reveals Differential Loss of Cholinergic Innervation in the Cortex and Hippocampus of the Adult Mouse Brain

**DOI:** 10.3389/fnagi.2019.00172

**Published:** 2019-07-04

**Authors:** Shereen Nizari, Roxana O. Carare, Ignacio A. Romero, Cheryl A. Hawkes

**Affiliations:** ^1^School of Life, Health and Chemical Science, Faculty of Science, Technology, Engineering and Mathematics, The Open University, Milton Keynes, United Kingdom; ^2^Clinical and Experimental Sciences, Faculty of Medicine, University of Southampton, Southampton, United Kingdom

**Keywords:** neurovascular unit, cholinergic, Alzheimer’s disease, cerebral amyloid angiopathy, cortex, hippocampus

## Abstract

Increasing evidence supports a role for cerebrovasculature dysfunction in the etiology of Alzheimer’s disease (AD). Blood vessels in the brain are composed of a collection of cells and acellular material that comprise the neurovascular unit (NVU). The NVU in the hippocampus and cortex receives innervation from cholinergic neurons that originate in the basal forebrain. Death of these neurons and their nerve fibers is an early feature of AD. However, the effect of the loss of cholinergic innervation on the NVU is not well characterized. The purpose of this study was to evaluate the effect of the loss of cholinergic innervation of components of the NVU at capillaries, arteries and veins in the hippocampus and cortex. Adult male C57BL/6 mice received an intracerebroventricular injection of the immunotoxin p75NTR mu-saporin to induce the loss of cholinergic neurons. Quadruple labeling immunohistochemistry and 3D reconstruction were carried out to characterize specific points of contact between cholinergic fibers and collagen IV, smooth muscle cells and astrocyte endfeet. Innate differences were observed between vessels of the hippocampus and cortex of control mice, including a greater amount of cholinergic contact with perivascular astrocytes in hippocampal capillaries and a thicker basement membrane in hippocampal veins. Saporin treatment induced a loss of cholinergic innervation at the arterial basement membrane and smooth muscle cells of both the hippocampus and the cortex. In the cortex, there was an additional loss of innervation at the astrocytic endfeet. The current results suggest that cortical arteries are more strongly affected by cholinergic denervation than arteries in the hippocampus. This regional variation may have implications for the etiology of the vascular pathology that develops in AD.

## Introduction

The loss of cholinergic neurons in the basal forebrain and the areas innervated by their fiber projections is a hallmark of Alzheimer’s disease (AD; Whitehouse et al., 1982; Francis et al., 1999). Decreased cholinergic innervation of the hippocampus and cortex is associated with memory impairment (Damasio et al., 1985), decreased mini-mental state examination scores and behavioral changes (Perry, 1980; Tong and Hamel, 1999; Garcia-Alloza et al., 2005). Moreover, acetylcholinesterase inhibitors (AChEIs) are currently one of only two approved drugs for the treatment of AD (Hampel et al., 2018). Recent studies have demonstrated that loss of basal forebrain gray matter occurs before the onset of clinical symptoms (Schmitz and Nathan Spreng, 2016) and that administration of the AChEI Donepezil during the prodromal stage of AD prevented basal forebrain degeneration (Cavedo et al., 2017). This highlights the significance of cholinergic neurotransmission in AD.

Numerous experimental models have been used to mimic the loss of basal forebrain cholinergic neurons and their fiber projections. These include injection of ibotenic acid into the substantia innominata (Vaucher and Hamel, 1995), lesioning of the fimbria fornix (van der Staay et al., 1989) and electric pulse ablation of the medial septum (Scheiderer et al., 2006; Nelson et al., 2014). However, these models can result in widespread degeneration that may not specifically target cholinergic cell populations. The discovery that cholinergic neurons in the basal forebrain express the p75 neurotrophin receptor (NTR), while other populations of cholinergic neurons do not (Steininger et al., 1993), allowed for the development of targeted immunotoxins such as 192 IgG-saporin and its mouse analog, mu-p75-saporin. *In vivo* administration of mu-p75-saporin has been shown to selectively kill cholinergic neurons in the medial septum, horizontal and diagonal bands of Broca and nucleus basalis of Meynert and cause withdrawal of cholinergic projections in the cortex and hippocampus in mice (Berger-Sweeney et al., 2001; Hunter et al., 2004; Hamlin et al., 2013; Kerbler et al., 2013; Laursen et al., 2013; Ramos-Rodriguez et al., 2013). Although recent genetically-driven technologies such as optogenetics and designer receptor exclusively activated by designer drug (DREDD) have led to more targeted approaches to silence specific cholinergic populations (Hangya et al., 2015; Zhang et al., 2017), it is not clear if these techniques replicate the loss of cholinergic innervation that is seen in AD.

In addition to early loss of cholinergic neurons, increasing evidence suggests that alterations of the cerebrovasculature contribute to the etiology and/or progression of AD. In fact, vascular pathology has been suggested to be the earliest indicator of the development of AD (Jack et al., 2010; Iturria-Medina et al., 2016). The most common form of cerebrovascular pathology associated with AD is cerebral amyloid angiopathy (CAA). CAA is defined as the presence of β-amyloid (Aβ) deposits in the walls of cerebral blood vessels (Vinters, 1987) and is believed to develop due to an age-related failure of clearance of Aβ from the brain. CAA develops principally in cortical and leptomeningeal arteries, with additional capillary involvement in individuals carrying the apolipoprotein E4 (apoE4) allele (Thal et al., 2008). It is observed least frequently in veins. Topographically, CAA starts in blood vessels of neocortical areas (e.g., occipital and parietal lobe), while subcortical vessels (e.g., hippocampus, thalamus) are typically not affected until later stages of the disease (Thal et al., 2008; Vinters and Gilbert, 1983). The reasons underlying the development of CAA and its pattern of distribution are currently unknown.

Blood vessels in the brain are composed of endothelial cells, basement membrane proteins, pericytes, smooth muscle cells, astrocytes and neurons that are collectively referred to as the neurovascular unit (NVU; Iadecola, 2017). The NVU is also a target of cholinergic innervation, which can occur at multiple sites, including astrocytes, smooth muscle cells and endothelial cells (Vaucher and Hamel, 1995). This innervation is important for the maintenance of vascular tone and in mediating site-directed blood flow *via* neurovascular coupling (Hamel, 2006). Loss of cholinergic contact with blood vessels has been reported in the cortex of AD brains (Tong and Hamel, 1999) and in transgenic mouse models of AD (Kuznetsova and Schliebs, 2013; Michalski et al., 2017). However, most of these studies have been carried out using 2D images and have focused on specific brain regions and/or selected vessels. Thus, the impact of the loss of cholinergic innervation on the entire NVU is not well characterized.

In this study, the mu-p75-saporin saporin model was used to induce death of basal forebrain cholinergic neurons and their fiber projections. Quadruple labeling immunohistochemistry and 3D reconstruction were carried out to characterize specific points of contact between cholinergic fibers and various components of the NVU and to compare this pattern between the cortex and the hippocampus.

## Materials and Methods

### Animals

Eight- to ten-week-old male C57BL/6 mice were obtained from The Open University (OU, Milton Keynes, UK) or the University of Southampton (Southampton, UK) and were kept on a 12 h light/dark cycle with access to food and water *ad libitum*. Experiments were carried out in compliance with guidelines of the Animal Welfare and Ethics Research Boards at the Open University and the University of Southampton and with approval from the Home Office (PPL 70/8507; PPL 30/3095).

### Intracerebroventricular Injections

Mice were anesthetized under isoflurane gas and placed into a stereotaxic frame (Kopf instruments, CA, USA). Topical anesthetic (Cryogesic, Acorus Therapeutics Limited Chester, UK) was applied to the scalp before the head was shaved. A midline incision was made and the skull cleaned. A small burr hole was drilled over the left and right lateral ventricles and 0.5 μL of mu-saporin (0.596 μg/μL, Advanced Targeting Systems, San Diego, CA, USA; *n* = 16) or 0.9% saline (*n* = 19) was injected into each ventricle (coordinates from Bregma: AP = −0.4 mm, ML = 1.0 mm, DV = −2.3 mm) at a rate of 0.2 μl/min using a 32G Hamilton syringe. The needle was left *in situ* for 2 min after the injection to allow for diffusion. Analgesia was administered intraperitoneally at the time of surgery (Carprieve, 5% w/v, 0.32 ml/kg, Norbrook, Northamptonshire, UK) and mice were able to self-administer sugar-free jelly (Hartley, Histon Sweet Spreads Limited, Leeds, UK) containing Carprofen (250 μg, Zoetis, London, UK) for 1 week post-surgery.

### Tissue Processing

All mice were perfused intracardially with 0.01 M phosphate buffered saline (PBS, pH 7.4) 45 days after surgery. For Western blots, brains were immediately dissected and snap frozen on dry ice and stored at −80°C. For immunohistochemistry, mice were perfused with 4% paraformaldehyde, the brains were post-fixed overnight and left in 30% sucrose for 1 week. Brains were cryosectioned at 20 μm thickness and collected as free-floating coronal sections and stored in anti-freeze storage solution (30% glycerol, 30% ethylene glycol, 40% 0.01 M PBS) at −20°C.

### Western Blotting

Tissues from control (*n* = 8) and saporin-treated mice (*n* = 6) were homogenized in Ripa lysis buffer [20 mM Tris-HCl (pH 8.0), 150 mM NaCl, 1 mM EDTA, 0.1% SDS, 1% Igepal, 50 mM NaF, 1 mM NaVO_3_] containing a protease inhibitor cocktail (Merck Millipore, Watford, UK), spun down (13,000 *g*, 10 mins, 4°C) and supernatants collected, aliquoted and frozen at −80°C until further use. Proteins (30 μg) were separated by gel electrophoresis on 4%–20% Tris-acetate gels (Fisher Scientific) and transferred onto a nitrocellulose membrane. Membranes were incubated overnight at 4°C with anti-choline acetyltransferase (ChAT, 1:500, Merck Millipore), stripped and re-probed with anti-glyceraldehyde-3-phosphate dehydrogenase (GAPDH, 1:50,000, Sigma-Aldrich, Dorset, UK) antibody to ensure equal protein loading. Two blots were replicated for each brain region. Immunoblots were quantified by densitometry using ImageJ software (NIH, MD, USA) and calculated as an optical density ratio of protein levels normalized to GAPDH levels.

### Immunohistochemistry

For single-labeling immunohistochemistry of cholinergic cell bodies and fibers, tissue sections were washed in 0.01 M PBS, blocked with 15% normal donkey serum (NDS; Sigma-Aldrich) and incubated overnight with either anti-ChAT (1:100) or anti-laminin (1:350, Sigma-Aldrich), after pre-treatment with pepsin (1 mg/mL in 0.2 N HCl, 30 s at 37°C). The next day, sections were washed in PBS and incubated for 2 h at room temperature with anti-donkey AlexaFluor 488 (Fisher Scientific, Loughborough, UK). For quadruple labeling of the NVU, sections were treated with pepsin, incubated overnight with anti-ChAT (1:100), washed in PBS and incubated with anti-goat AlexaFluor 555. After washing in PBS, sections were incubated simultaneously with anti-collagen IV (1:100, Abcam, Cambridge, UK), anti-α smooth muscle actin (α-SMA)-FITC (1:350, Sigma-Aldrich) and anti-glial fibrillary protein (GFAP, 1:500, Abcam). Sections were then developed with anti-rabbit AlexaFluor 405 and anti-chicken AlexaFluor 633 (1:200, Fisher Scientific). All fluorescent sections were coverslipped using Mowiol^®^ (Sigma-Aldrich) containing 0.1% v/v Citifluor (Citifluor Limited, London, UK) mounting media.

### Image Acquisition and Analysis

Coronal brain sections were imaged with an SP5 Leica scanning laser confocal microscope. Low magnification images of the cortex and hippocampus were stitched together using ImageJ software (NIH, MD, USA). The density of neuronal cell bodies, fibers and blood vessels in each region of interest was quantified by calculating the percentage area covered by staining using ImageJ software. NVUs in the hippocampus and cortex were imaged using the ×100 oil immersion objective, using *z* stacks with ≤2 μm spacing between slices. Images were deconvolved and converted into Imaris-compatible files using AutoQuant X3 version X3.0.4 software (MediaCybernetics Inc., Rockville, MD, USA).

### 3D Reconstruction of the NVU

To quantify the parameters of each component of the NVU, deconvolved images were processed using Imaris software (Bitplane^®^) and surfaces were created for each component of the NVU. For each vessel, the following measurements were acquired: the total area of a selected surface (μm^2^), the volume of a selected surface (μm^3^), the length of the vessel imaged (μm) and the average diameter of the vessel (μm). The total area of contact between two selected surfaces (e.g., ChAT nerve fibers contacting collagen IV) was calculated using the Imaris Xtension “Surface to Surface Contact Area” (Imaris V8.31, ImarisXT Bitplane Inc created by Matthew J Gastinger, Bitplane). Only surfaces that made direct contact with each other (i.e., 0 μm apart) were quantified. Vessels were classified as capillaries if they were ≤10 μm in diameter, arteries were identified as having a diameter of >10 μm and positive for SMA, while veins were identified as having a diameter >10 μm but lacking SMA. A total of five capillaries, five arteries and three veins were quantified for each mouse (*n* = 7–11 control, *n* = 6–10 saporin) per brain region and the average values per mouse were used for statistical analysis.

### Statistical Analysis

Data were tested for normality using the Kolmogorov-Smirnov test. For normally distributed data, comparisons between two groups were carried out using two-tailed Student’s *t*-test. Where there were more than two groups, one-way or two-way repeated measures ANOVA was used followed by Sidak’s *post hoc*. The ROUT test was used to identify and exclude any outliers. For data that were not normally distributed, the Mann-Whitney *U* test or Kruskal-Wallis test with Dunn’s *post hoc* test was used. Data represents mean ± SEM and *p* < 0.05 was considered to be statistically significant. Analysis was carried out using GraphPad Prism software.

## Results

### Cholinergic Loss in the Medial Septum, Hippocampus and Cortex Following Administration of Mu-Saporin

**Figure 1:**
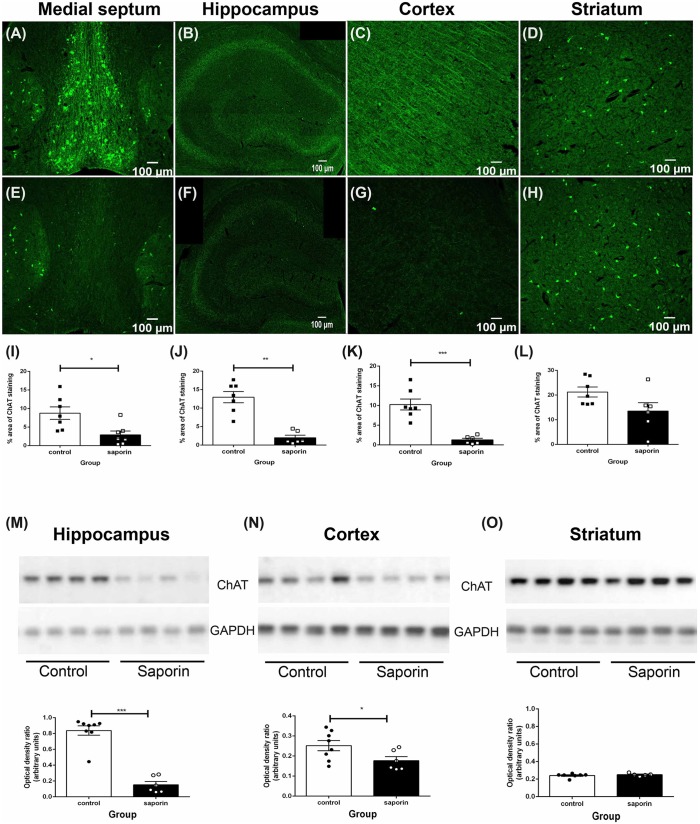
Loss of cholinergic neurons and fiber projections following administration of mu-p75-saporin.** (A–H)** Photomicrographs of choline acetyltransferase (ChAT)-positive neurons and fibers in the medial septum **(A,E)**, hippocampus **(B,F)**, cortex **(C,G)** and striatum **(E,H)** in control **(A–D)** and saporin-treated **(E–H)** mice. **(I–L)** Quantification of the percent area covered by ChAT-positive staining in control and saporin-treated mice in the medial septum **(I)**, hippocampus **(J)** and cortex **(K)**. p75-negative cholinergic neurons in the striatum were not affected by saporin treatment **(L)**. **(M–O)** Western blotting confirmed a significant loss of ChAT protein expression in the hippocampus **(M)** and cortex **(N)** after saporin administration, while ChAT levels in the striatum did not differ between control and treated mice **(O)**. Data represent mean ± SEM. *p < 0.05, **p < 0.01, ***p < 0.001, two-tailed Student’s *t*-test. Scale bar = 100 μm.

As shown in Figure 1, ChAT-positive cholinergic neurons were observed in the medial septum, diagonal band of Broca, nucleus basalis of Meynert and in the striatum of control mice. Cholinergic fiber projections were also observed in the hippocampus (Figure 1B) and cortex (Figure 1C). Significantly less ChAT staining was detected in the medial septum of saporin-treated mice at 45 days post-surgery (Figures 1E,I). This was accompanied by a significant decrease in cholinergic nerve fiber density in the hippocampus (Figures 1F,J) and the cortex (Figures 1G,K). As expected, p75 NTR-negative neurons in the striatum were not affected by saporin treatment (Figures 1H,L). Western blotting confirmed a significant decrease in ChAT protein levels in the hippocampus (Figure 1M) and cortex (Figure 1N) following saporin administration and no difference in ChAT expression between control and saporin-treated mice in the striatum (Figure 1O).

### Characterization of Cholinergic Loss at the NVU in the Hippocampus and Cortex

**Figure 2:**
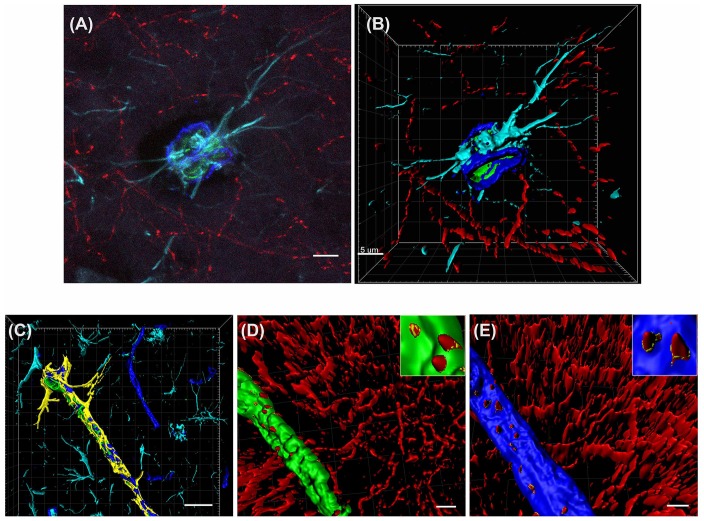
3D reconstruction of the neurovascular unit (NVU). **(A,B)** Photomicrograph **(A)** and 3D reconstruction **(B)** of an artery stained for collagen IV (dark blue), smooth muscle actin (SMA; green), astrocytes (turquoise) and cholinergic nerve fibers (red). **(C–E)** Yellow outlines indicate the surfaces created for each of the NVU components. Examples are shown for surface area contact between perivascular astrocytes and collagen IV **(C)**, contact of cholinergic nerve fibers to smooth muscle cells (**D**, yellow outlines in inset) and contact of cholinergic nerve fibers to collagen IV (**E**, yellow outlines in inset). Scale bar **(A,B,D,E)** = 5 μm, **(C)** = 20 μm.

Cholinergic nerve fibers are known to innervate blood vessels in the hippocampus and cortex (Vaucher and Hamel, 1995). To characterize the precise effects of saporin treatment at the NVU, quadruple-labeling immunohistochemistry was used to label cholinergic fibers and three components of the NVU—collagen IV-positive basement membranes, smooth muscle cells and astrocytes (Figure 2A). Confocal images of the vessels were then reconstructed using 3D modeling software and surfaces were created for each of the four proteins (Figures 2B–E). The surface area of ChAT contact with each component of the NVU (standardized to vessel length) was analyzed across capillaries, arteries/arterioles and veins/venules from control and saporin-treated mice.

**Figure 3:**
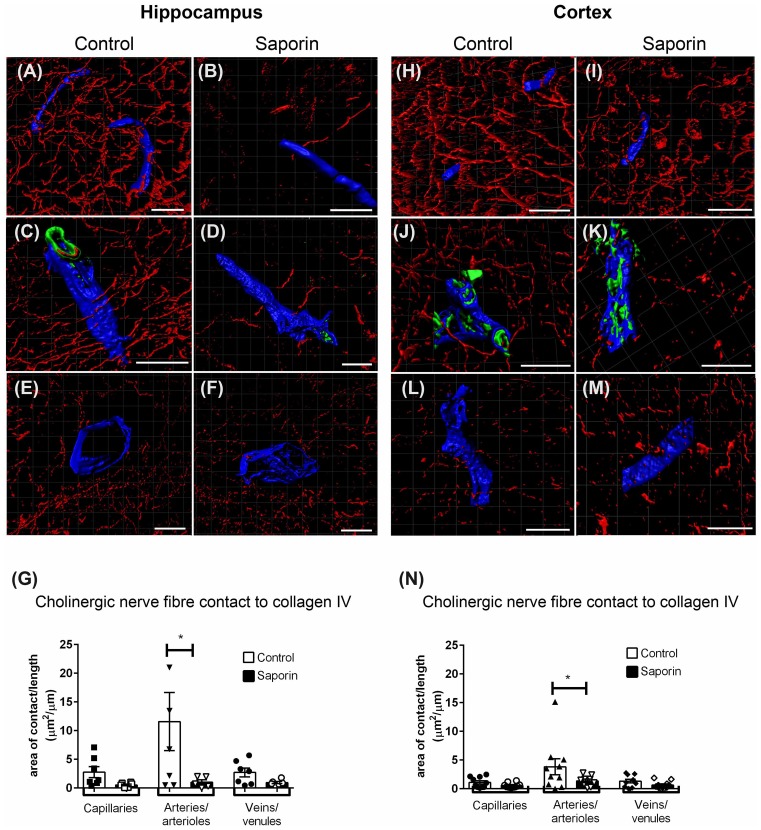
Perivascular cholinergic contact at the basement membrane.** (A–M)** Representative images of the 3D reconstruction of the NVU in capillaries **(A,B,H,I)**, arteries **(C,D,J,K)** and veins **(E,F,L,M)** in the hippocampus **(A–F)** and cortex **(H–M)** of control **(A,C,E,H,J,L)** and saporin-treated mice **(B,D,F,I,K,M)**. ChAT-positive fibers are shown in red, collagen IV is shown in blue and SMA is shown in green. **(G,N)** Quantification of the surface area of contact between ChAT-positive fibers and collagen IV demonstrated a significant decrease in contact at the arteries of saporin-treated animals in both the hippocampus **(G)** and cortex **(N)**. Data represent mean ± SEM. *p < 0.05, two-way ANOVA with Sidak’s *post hoc* test. Scale bars = 20 μm.

At the basement membrane of vessels in the hippocampus, saporin treatment induced a decrease in the amount of contact between cholinergic nerve fibers and collagen IV at capillaries, arteries and veins (Figures 3A–F), although this decrease was only statistically significant at arteries (Figure 3G). In the cortex, cholinergic innervation of the basement membrane did not differ between control and saporin-treated mice at capillaries and veins but was significantly decreased at the arteries of saporin-treated mice (Figures 3H–N). No differences were noted between control and saporin-treated mice at any vessel type in the striatum (Supplementary Figure S1A).

**Figure 4:**
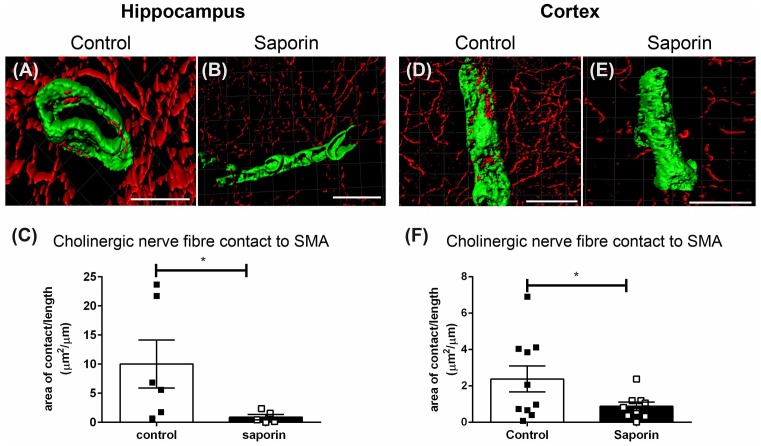
Perivascular cholinergic contact at smooth muscle cells. **(A–E)** Representative images of the 3D reconstruction of the NVU in arteries in the hippocampus **(A,B)** and cortex **(D,E)** from control **(A,D)** and saporin-treated **(B,E)** mice. ChAT-positive nerve fibers are shown in red and smooth muscle cells are shown in green. **(C,F)** Quantification of the surface area of contact between ChAT-positive fibers and SMA demonstrated a significant decrease in contact in saporin-treated animals in both the hippocampus **(C)** and cortex **(F)**. Data represent mean ± SEM. *p < 0.05, Mann-Whitney *U* test (hippocampus) and one-tailed Student’s *t*-test (cortex). Scale bars = 20 μm.

Analysis of perivascular innervation at the smooth muscles of arteries found that there was a significant decrease in the surface area contact between ChAT and α-SMA in saporin-treated in both the hippocampus (Figures 4A–C) and the cortex (Figures 4D–F), while the striatum was not affected (Supplementary Figure S1B).

**Figure 5:**
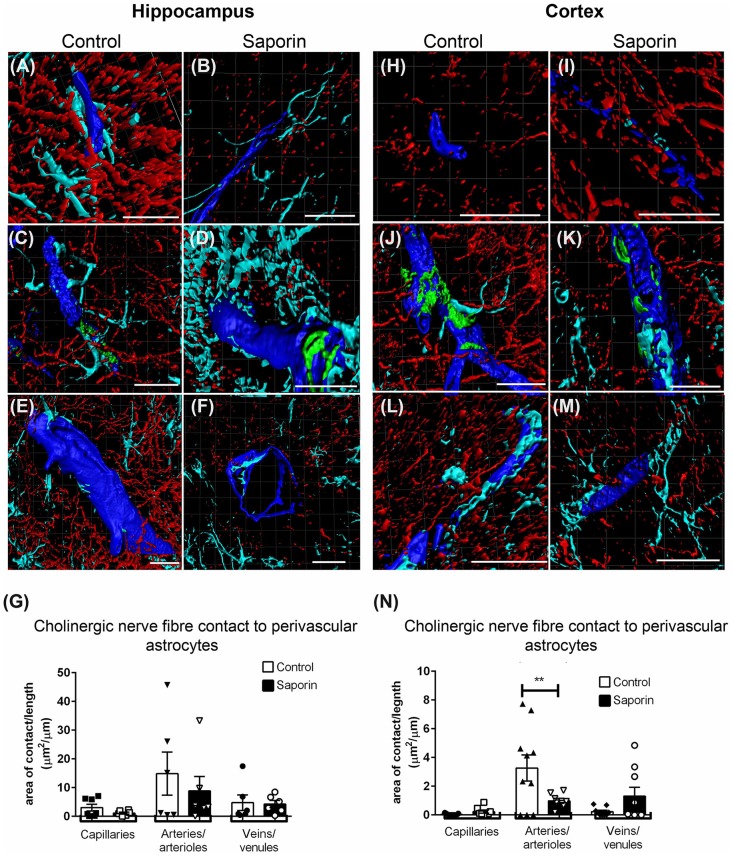
Perivascular cholinergic contact at perivascular astrocytes.** (A–M)** Representative images of the 3D reconstruction of the NVU in capillaries **(A,B,H,I)**, arteries **(C,D,J,K)** and veins **(E,F,L,M)** in the hippocampus **(A–F)** and cortex **(H–M)** of control **(A,C,E,H,J,L)** and saporin-treated mice **(B,D,F,I,K,M)**. ChAT-positive fibers are shown in red, collagen IV is shown in blue, SMA is shown in green and glial fibrillary protein (GFAP) is shown in turquoise. **(G,N)** Quantification of the surface area of contact between ChAT-positive fibers and astrocyte endfeet found no differences between control and saporin-treated animals in any vessels of the hippocampus **(G)**, but a significant decrease in contact at the arteries of saporin-treated animals the cortex **(N)**. Data represent mean ± SEM. **p < 0.01, two-way ANOVA with Sidak’s *post hoc* test. Scale bars = 20 μm.

Quantification of ChAT contact with perivascular astrocytes in the hippocampus revealed no difference in the amount of contact with GFAP-positive astrocytes between control and saporin-treated mice in any vessel type (Figures 5A–G). By contrast, significantly less ChAT contact was observed at arteries in the cortex of saporin-treated mice compared to control animals (Figures 5H–N). No differences were observed between control and saporin mice at cortical capillaries or veins (Figures 5H–N) or between control and saporin-treated mice at any vessel type in the striatum (Supplementary Figure S1C).

**Figure 6:**
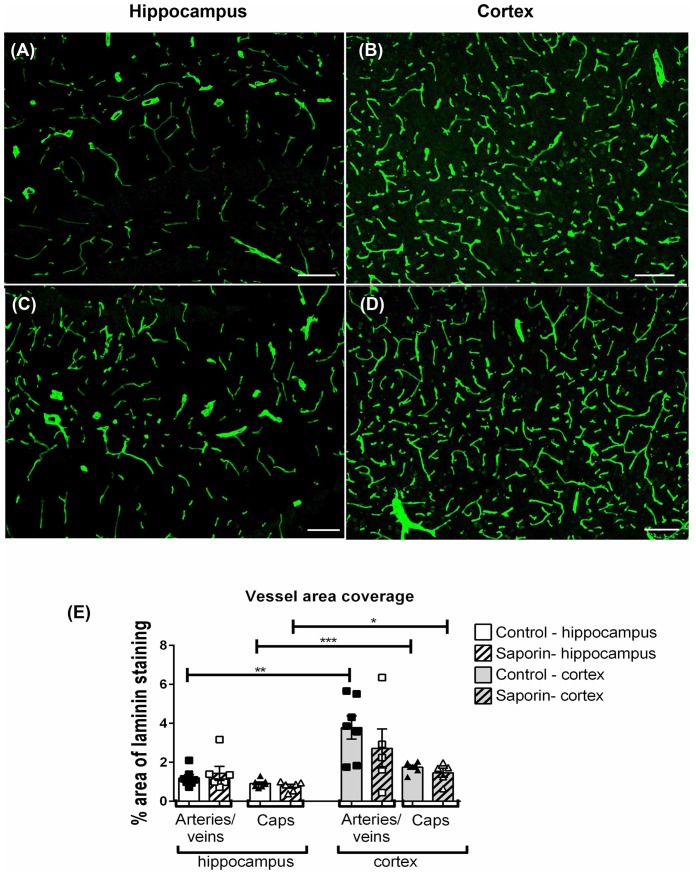
Regional comparison of blood vessel density.** (A–D)** Photomicrographs of laminin-positive blood vessels in the hippocampus **(A,C)** and cortex **(B,D)** of control **(A,B)** and saporin-treated mice **(C,D)**. **(E)** Quantification of the density of capillaries (caps) and large-diameter vessels in the hippocampus and cortex in treatment groups. Data represent mean ± SEM. *p < 0.05, **p < 0.01, ***p < 0.001, two-tailed Student’s *t*-test. Scale bar = 100 μm.

To determine if saporin induced changes in blood vessel density, the percent area covered by laminin-positive capillaries and large-diameter vessels were quantified in the cortex and hippocampus of control and saporin-treated mice (Figures 6A–E). In control mice, the density of both capillaries and arteries/veins was significantly higher in the cortex compared to the hippocampus (Figures 6A,B). No differences in vessel density were noted between control and saporin-treated mice in either brain region (Figure 6E).

### Regional Variation in Perivascular Innervation

**Figure 7:**
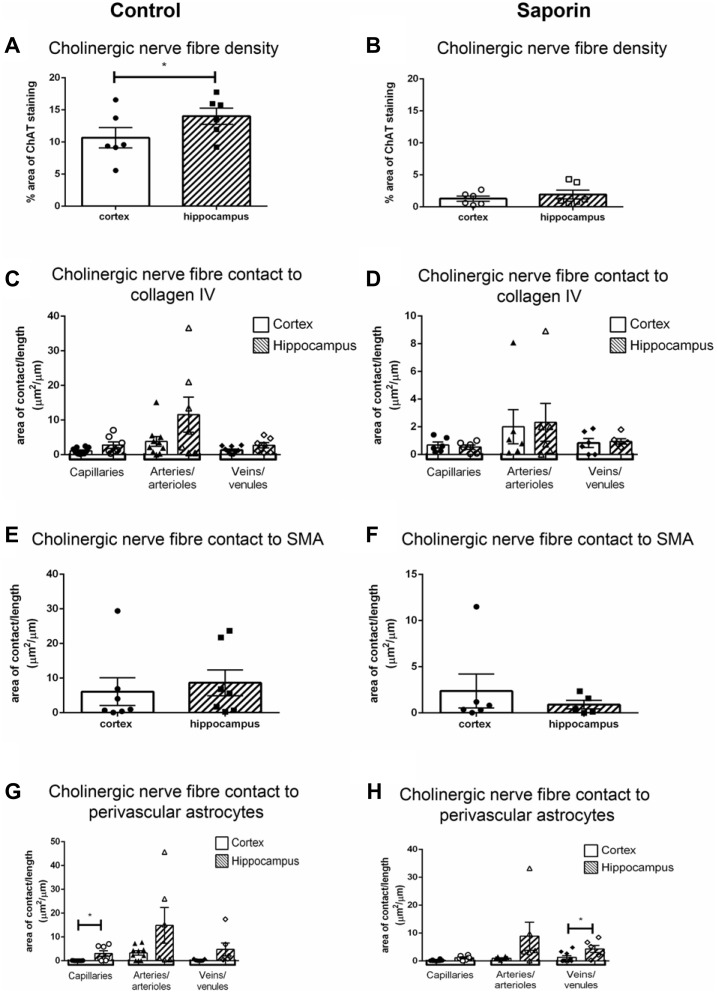
Regional comparison of measures of the NVU. **(A,B)** Total density of ChAT-positive fibers in the cortex and hippocampus in control mice **(A)** and following administration of saporin **(B)**. **(C–H)** Quantification of area of contact between ChAT-positive fibers and collagen IV **(C,D)**, SMA **(E,F)** and astrocyte endfeet **(G,H)** in the cortex and hippocampus in control **(C,E,G)** and saporin-treated mice **(D,F,H)**. Data represent mean ± SEM. *p < 0.05, paired two-tailed Student’s *t*-test **(A,B)**, Kruskal-Wallis with Dunn’s *post hoc* test, **(C,D)**, Wilcoxon matched paired test **(E–H)**.

To determine if there were regional differences in endogeneous and saporin-induced cholinergic innervation, measurements of ChAT contact with components of the NVU were compared between the hippocampus and cortex. Quantification of overall cholinergic nerve fiber density was found to be significantly higher in the hippocampus compared to the cortex in control mice (Figure 7A). Following saporin treatment, this difference was lost (Figure 7B). The degree of cholinergic innervation at the basement membrane did not differ between the hippocampus and cortex at capillaries, arteries or veins of control (Figure 7C) or saporin-treated mice (Figure 7D). ChAT innervation of smooth muscle cells also did not differ between arteries in the hippocampus and cortex in either control (Figure 7E) or saporin-treated mice (Figure 7F). However, perivascular astrocyte endfeet surrounding cortical capillaries in control mice received significantly less cholinergic input compared to astrocytes at the capillaries in the hippocampus (Figure 7G). Significantly less cholinergic input onto astrocyte endfeet was also noted at veins in the cortex of saporin-treated mice compared to hippocampal veins (Figure 7H).

### Impact of Loss of Perivascular Innervation on Components of the NVU

To determine if loss of cholinergic innervation induced changes in components of the NVU, the volume of collagen IV and smooth muscle cells as well as the area of astrocyte endfoot coverage was evaluated in vessels of control and saporin-treated mice. Comparisons between the cortex and hippocampus were also carried out.

As shown in Table 1, within the hippocampus, the volume of collagen IV was significantly higher in veins compared to capillaries in control mice. This relationship was maintained following saporin treatment. However, collagen IV volumes did not differ between control and saporin-treated mice in any vessel type. In the cortex, the volume of collagen IV was highest in arteries compared to capillaries and veins in both control and saporin-treated mice (Table 1). Regional comparisons including the striatum revealed that the volume of collagen IV was significantly higher in veins in the hippocampus than veins in the cortex and striatum in both control mice and those treated with saporin.

**Table 1:** Regional comparison of volume of basement membrane across vessel type.

Basement membrane Mean volume/length (μm^3^/μm) ± SEM	Hippocampus			Cortex			Striatum		
	Capillaries	Arteries	Veins	Capillaries	Arteries	Veins	Capillaries	Arteries	Veins
Control	32.63 ± 7.9*	87.49 ± 22.6	148.9 ± 40.6*^▴^	18.82 ± 3.4^▿^	114.5 ± 40.3^▿^	42.68 ± 7.7^▴^	26.82 ± 3.6^▿^	106.7 ± 29.2^▿^	51.05 ± 6.7^▴^
Saporin	32.71 ± 5.4*	114.7 ± 22.2	153.8 ± 30.3*^▴^	21.26 ± 2.4	84.56 ± 11.5^∞^	37.38 ± 7.2^▴∞^	21.95 ± 1.6^▿^	73.46 ± 18.3^▿^	35.45 ± 2.8^▴^

Data represents mean ± SEM. *p < 0.05 capillaries vs. veins; ^▿^p < 0.05 capillaries vs. arteries; ^∞^p < 0.05 arteries vs. veins (one-way ANOVA with Sidak’s post hoc) ^▴^p < 0.01; hippocampus vs. cortex or striatum (two-way repeated measures ANOVA with Sidak’s post hoc analysis).

Analysis of smooth muscle volume found no significant difference between control and saporin mice in any brain region (Table 2). Similarly, no significant differences in smooth muscle volume were noted between the cortex, hippocampus or striatum in either treatment group (Table 2).

**Table 2:** Regional comparison of volume of smooth muscle cells in arteries.

SMA Volume/length (μm^3^/μm) ± SEM	Hippocampus	Cortex	Striatum
Control	60.02 ± 10.6	50.75 ± 16.2	65.88 ± 19.7
Saporin	33.10 ± 7.4	39.54 ± 9.0	33.03 ± 5.7

*Values represent mean ± SEM. No significant differences were noted (two-way repeated measures ANOVA with Sidak’s post hoc test)*.

Finally, analysis of perivascular astrocyte coverage of hippocampal vessels revealed a significantly higher amount of endfoot contact in veins compared to capillaries in control animals (Table 3). Following saporin administration, astrocyte coverage remained significantly higher in hippocampal veins compared to both capillaries. No differences in perivascular astrocyte coverage were noted between vessel types in the cortex in either control or saporin-treated mice (Table 3). Comparison between vessels of the hippocampus, cortex and striatum found that astrocyte contact was lowest in all the vessel types in the striatum. In addition, astrocyte coverage of veins in the hippocampus was approximately 10-fold higher compared to coverage of veins in the cortex and 100-fold higher than veins in the striatum in both control and saporin mice (Table 3).

**Table 3:** Area of astrocyte coverage of basement membrane.

Astrocyte endfoot coverage of basement membrane. Area of contact/length (μm^2^/μm) ± SEM	Hippocampus			Cortex			Striatum		
	Capillaries	Arteries	Veins	Capillaries	Arteries	Veins	Capillaries	Arteries	Veins
Control	6.7 ± 2.1*	38.43 ± 8.8^▴^	81.84 ± 21.4*^▴^	2.23 ± 1.5	23.72 ± 13.8	10.24 ± 6.1^▴^	0.32 ± 0.2	0.357 ± 0.08^▴^	0.457 ± 0.2^▴^
Saporin	15.26 ± 3.5*	52.54 ± 12^▴^	84.35 ± 14.4*^▴^	4.22 ± 1.8	21.05 ± 6.6	8.57 ± 5.3^▴^	0.317 ± 0.2	0.105 ± 0.03^▴^	0.121 ± 0.06^▴^

Values represent mean ± SEM. *p < 0.05 capillaries vs. veins (two-way repeated measures ANOVA with Sidak’s post hoc) ^▴^p < 0.05; hippocampus vs. cortex or striatum (two-way repeated measures ANOVA with Sidak’s post hoc).

## Discussion

Although the loss of cholinergic neurons in AD has been described extensively, the effect of this loss on the NVU is not well characterized. Moreover, previous studies that have looked at perivascular innervation by cholinergic nerve fibers have largely been carried out using 2D images obtained from double or triple labeling immunohistochemistry or by immuno-EM (Itakura et al., 1977; Tong and Hamel, 1999; Kuznetsova and Schliebs, 2013). By combining quadruple labeling immunohistochemistry with 3D reconstruction, the current study allowed not only for individual components of the NVU to be assessed under basal and pathological conditions but also for perivascular innervation to be quantified along multiple components of the NVU around the entire surface of the blood vessel.

We chose to use the saporin model of cholinergic denervation because of its well characterized ability to cause selective death of basal forebrain cholinergic neurons across many species, including mice, rats and non-human primates (Fine et al., 1997; Leanza, 1998; Lin et al., 1999; Berger-Sweeney et al., 2001; Lehmann et al., 2002; Birthelmer et al., 2003; Hawkes et al., 2005; Scheiderer et al., 2006; Ramos-Rodriguez et al., 2013). Such immunotoxin models are preferable to older lesioning models that can result in widespread, non-specific neuronal damage (van der Staay et al., 1989; Scheiderer et al., 2006; Nelson et al., 2014). On the other hand, the relatively rapid time course of death induced by saporin is unlikely to mimic the progressive loss of cholinergic neurons that is seen in AD. More refined methods have been developed to silence basal forebrain cholinergic neurons using optogenetics and DREDD technologies (Shi et al., 2015; Chen et al., 2016). However, whether such techniques fully replicate the loss of cholinergic signaling, including withdrawal of trophic support and related inflammatory processes, that is observed in AD is not yet known. In the present study, administration of saporin led to a significant and specific loss of cholinergic neurons in the basal forebrain at 45 days post-surgery. The fiber projections from these neurons were also lost in the hippocampus and cortex. Other major populations of cholinergic neurons that do not express the p75NTR, including those in the striatum (Yeo et al., 1997) were not affected by saporin treatment.

Using 3D reconstruction of blood vessels, ChAT-positive fibers were found to innervate capillaries, arteries and veins in the hippocampus and cortex. As expected (Toribatake et al., 1997; Mulligan and MacVicar, 2004; Hamel, 2006; Hamilton et al., 2010; Chen et al., 2014), cholinergic innervation was observed at all levels of the NVU investigated, including the basement membranes, smooth muscle cells and perivascular astrocytes. The majority of the innervation was observed at arteries, in agreement with previous studies (Chédotal et al., 1994; Vaucher and Hamel, 1995; Luiten et al., 1996; Kuznetsova and Schliebs, 2013). Predominant targeting of arteries by cholinergic nerve fibers is perhaps unsurprising given the role of ACh in mediating neurovascular coupling (Hamel, 2006; Willis et al., 2006; Lecrux et al., 2017).

Increasing evidence suggests that there is heterogeneity of cells of the NVU, including pericytes and astrocytes, across both vessel type and brain regions (Shepro and Morel, 1993; Noumbissi et al., 2018). In the present study, we observed innate differences between vessels of the hippocampus and cortex. These included: (i) a significantly higher vessel density in the cortex; (ii) a higher overall ChAT fiber density in the hippocampus and more ChAT contact with perivascular astrocytes in hippocampal capillaries; (iii) thicker basement membrane in the veins of the hippocampus; and (iv) greater coverage of the basement membrane by astrocyte endfeet in hippocampal veins compared to veins in the cortex.

The observation that total cholinergic fiber density was higher in the hippocampus compared to the cortex is in agreement with previous reports (Kitt et al., 1994). However, given that vesssel density showed the opposite pattern, it is perhaps surprising that there was no difference in the amount of cholinergic innervation at blood vessels in the hippocampus vs. those in the cortex. It is possible that by only quantifying direct contact (e.g., 0 μm distance) between ChAT fibers and basement membrane or smooth muscle cells, we have underestimated the potential degree of cholinergic innervation at the NVU, which has been classified in previous studies to be within 3 μm from the basement membrane (Vaucher and Hamel, 1995). Our findings that cholinergic contact with perivascular astrocyte endfeet tended to be higher in the hippocampus across all vessel types and was significantly higher at hippocampal capillaries compared to cortical capillaries, suggest that there may be functional differences between cortical and hippocampal vessels in their responsiveness to cholinergic signaling.

Astrocyte coverage of vessels was also observed to be higher in the hippocampus than the cortex, although this was only significant at veins. This is in keeping with the reported distribution of parenchymal GFAP-positive astrocytes (Emsley and Macklis, 2006). This finding was likely related to the observed greater thickness of collagen IV, given that astrocytes and endothelial cells are the main sites of basement membrane production (Baeten and Akassoglou, 2011). Expression of collagen IV has been shown to be significantly upregulated in capillaries and arteries during normal and in AD (Kalaria and Pax, 1995; Farkas and Luiten, 2001; Christov et al., 2008; Magaki et al., 2018). Thickening of the basement membrane and alterations in basement membrane composition has been hypothesized to precede the development of CAA (Wyss-Coray et al., 2000). However, veins are the vessel type least likely to be affected by CAA and CAA develops more slowly in vessels in the hippocampus than those in the cortex (Thal et al., 2008). It may be that increased basement thickness makes the veins in the hippocampus less likely to be deformed by pressure changes and thus helps to ensure a consistent cerebral perfusion (Zócalo et al., 2013; Thorin-Trescases et al., 2018) and to maintain a driving force for clearance of solutes in the cerebral spinal fluid (CSF) and/or interstitial fluid (ISF). In addition, the walls of veins are important for the egress of leukocytes from the blood into the brain in neurodegenerative diseases and this process requires that leukocytes enter a perivenular space bounded by endothelial and glia limitans basement membranes (Owens et al., 2008; Engelhardt et al., 2016). The variation in the degree of collagen IV and astrocyte coverage may reflect regional variability in the neuroinflammatory properties of the veins in the hippocampus compared to the cortex.

Saporin treatment significantly reduced the amount of cholinergic contact with the basement membrane of arteries in both the cortex and hippocampus, while capillaries and veins were unaffected. This may reflect the proportional endogeneous degree of cholinergic innervation between vessel types, which was highest in arteries. Regional differences were also observed in the degree of cholinergic loss at the NVU. While saporin treatment induced a loss of cholinergic innervation at the basement membrane and smooth muscle cells of arteries in both the hippocampus and cortex, there was additional loss of cholinergic contact of astrocyte endfeet in cortical arteries. The reason for this variability is unknown. It may be that cholinergic supply of the cortical astrocytes is important for their function in the convective influx/glymphatic entry of CSF along the pial glial basement membranes (Albargothy et al., 2018). Recent 3D mapping studies have shown that the dendritic arbors of basal forebrain neurons that project to the cortex differ from those that project to the hippocampus in that single cortical dendrites innervate large areas of the neuropil (Wu et al., 2014; Li et al., 2018). It is possible that a similar pattern of innervation exists at blood vessels in the cortex such that the loss of one dendritic arbor affects multiple vessels. This may also be related to the lower endogeneous level of contact between cholinergic nerves and astrocytes in the cortex, which may make cortical vessels more susceptible than those in the hippocampus to loss of cholinergic innervation.

Each of the NVU components studied have been shown to play a role in mediating the clearance of Aβ from the brain. Cerebrovascular basement membranes act as conduits along which Aβ contained within CSF and ISF is removed from the brain (Iliff et al., 2012; Hawkes et al., 2013; Morris et al., 2014; Albargothy et al., 2018). Smooth muscle cells express low-density receptor related protein-1 (LRP-1) which mediates cellular uptake of Aβ and its transcytosis across the blood brain barrier (BBB; Kanekiyo et al., 2012). Moreover, localized contraction of smooth muscles has recently been proposed to generate the force that drives intramural periarterial drainage of Aβ (Aldea et al., 2019). Astrocytes contribute to the formation of the basement membrane and have also been shown to take up Aβ *via* LRP-1 (Basak et al., 2012). In addition, astrocytes are the main producers of apolipoprotein E, which chaperones Aβ across the BBB (Bell et al., 2007). Therefore, it is possible that the combined loss of cholinergic innervation at each of these components contributes to the increased susceptibility of cortical vessels to the development of CAA. Further studies are needed to investigate this putative relationship in human brain tissues.

## Data Availability

The datasets generated for this study are available on request to the corresponding author.

## Ethics Statement

This study was carried out in accordance with the recommendations of the Animal Welfare and Ethics Research Boards at the Open University and the University of Southampton. The protocol was approved by the Home Office (PPL 70/8507; PPL 30/3095).

## Author Contributions

SN performed the experiments and data analysis. RC, IR and CH planned the experimental design. SN and CH wrote the manuscript.

## Conflict of Interest Statement

The authors declare that the research was conducted in the absence of any commercial or financial relationships that could be construed as a potential conflict of interest.
