# Supplementary material for: 3D Reconstruction of the Neurovascular Unit Reveals Differential Loss of Cholinergic Innervation in the Cortex and Hippocampus of the Adult Mouse Brain
Source: Front Aging Neurosci. 2019 Jul 4;11:172. doi: 10.3389/fnagi.2019.00172 (PMC6620643; doi:10.3389/fnagi.2019.00172)
Supplement: Supplementary file 1 [file Table_1.DOCX]

**Supplemental Figure 1 Measures of the NVU in the striatum:** (A-C) Quantification of area of contact between ChAT-positive fibers and collagen IV (A), smooth muscle actin (SMA, B) and astrocyte endfeet (C) in the striatum of control and saporin-treated mice. Data represent mean ±SEM.
