# Supplementary figures and images for: 3D Reconstruction of the Neurovascular Unit Reveals Differential Loss of Cholinergic Innervation in the Cortex and Hippocampus of the Adult Mouse Brain
Source: Front Aging Neurosci. 2019 Jul 4;11:172. doi: 10.3389/fnagi.2019.00172 (PMC6620643; doi:10.3389/fnagi.2019.00172)

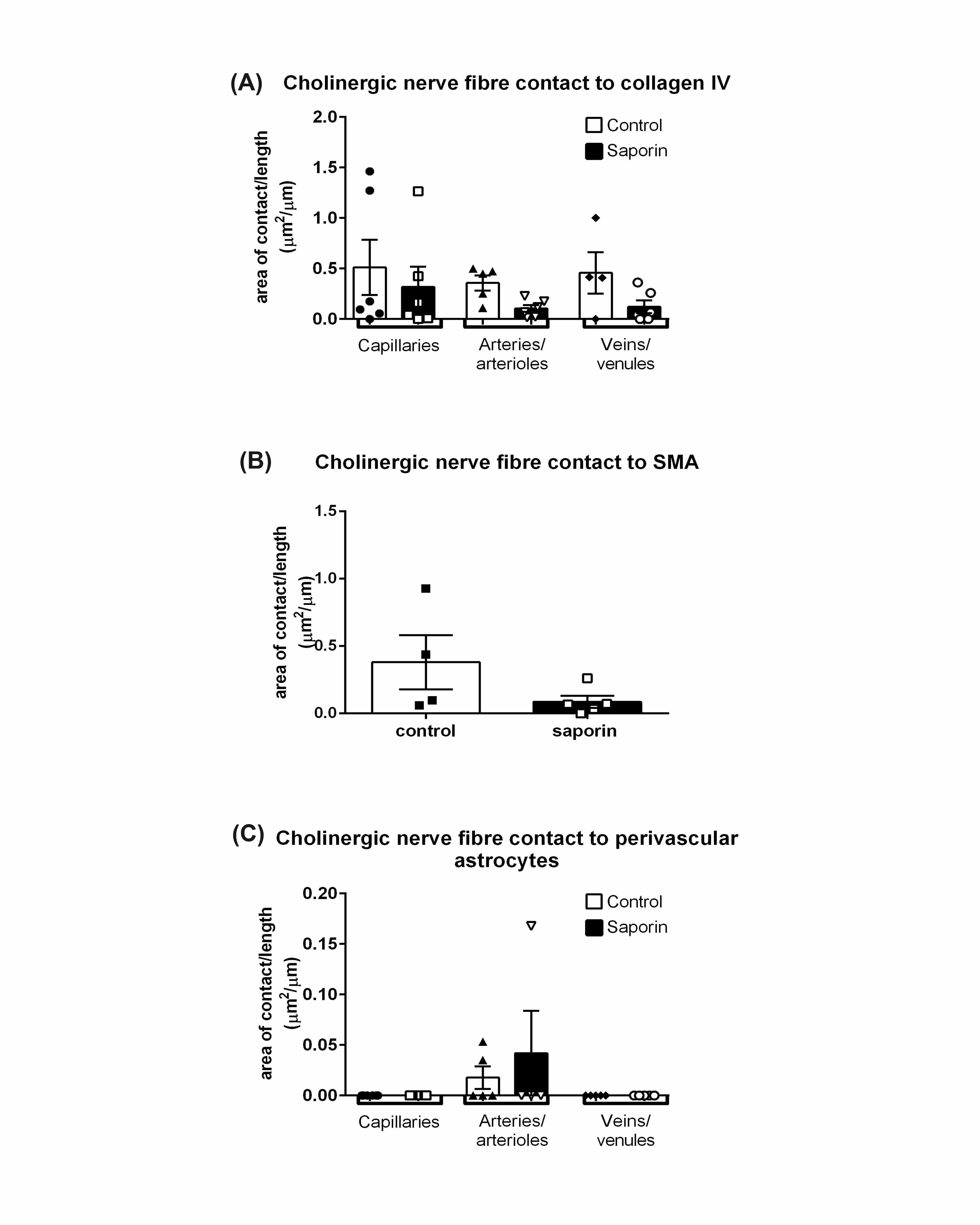

Supplement: Supplementary file 2 [file Image_1.JPEG]
